# Enzymes in biosynthesis

**DOI:** 10.3762/bjoc.18.116

**Published:** 2022-08-30

**Authors:** Jeroen S Dickschat

**Affiliations:** 1 Kekulé-Institut für Organische Chemie und Biochemie, Rheinische Friedrich-Wilhelms-Universität Bonn, Gerhard-Domagk-Straße 1, 53121 Bonn, Germany

**Keywords:** biosynthesis, enzymes in biosynthesis

Enzymes are fascinating biocatalysts that can accelerate remarkable transformations in nature. Some of the most interesting transformations catalyzed by enzymes are known from the biosynthetic pathways towards natural products. For instance, class I terpene synthases can convert highly complex transformations of an acyclic precursor, such as farnesyl or geranylgeranyl diphosphate, into sesqui- or diterpenes, respectively. As has been described recently, even farnesylfarnesyl diphosphate can be converted into triterpenes, a substance class that was previously believed to originate exclusively from squalene by class II terpene synthases [1]. These conversions proceed through multistep cationic cascade reactions and usually produce a polycyclic terpene hydrocarbon or alcohol with multiple stereogenic centers. While these transformations require only a single enzyme, polyketide and nonribosomal peptide biosyntheses are catalyzed by megasynthases that follow an assembly line logic, with individual domains for each single step [2]. Furthermore, the domains are organized into modules, each of which is responsible for the incorporation of one extender unit into the growing polyketide or peptide chain. With our knowledge today, the function of these large enzyme factories is easier to read than for terpene synthases, the functions of which are difficult to predict, but their size makes the megasynthases much more difficult to handle in the laboratory. Besides these core enzymes of the biosynthetic machineries to some of the most important classes of natural products, nature has evolved a large number of enzyme classes for more specific transformations, including cytochromes P450 or α-ketoglutarate-dependent dioxygenases for late-stage oxidations and transferases for the attachment of sugar units, acyl, or methyl groups. Moreover, some enzymes can catalyze reactions that were first known from synthetic chemistry, e.g., pericyclases can promote pericyclic reactions such as [4 + 2]-cycloaddition, also known as Diels–Alder reaction [3]. In fact, most named reactions in organic chemistry originally discovered by synthetic chemists have an analogy in nature, requiring a sophisticated enzymology [4]. Recent developments show us that there is still much more to discover, e.g., altemicidin was shown to be enzymatically constructed from NAD^+^ and SAM that usually serve as enzyme cosubstrates in redox transformations and methylations but are rarely used to construct the molecular scaffolds of natural products [5].

During the past two decades, large amounts of genome information from thousands of organisms have become available. This allows scientists today to gain direct access to the encoded catalysts through expression in easy-to-handle heterologous hosts, such as *Escherichia coli* or *Saccharomyces cerevisiae*. Besides in vitro studies with purified enzymes, heterologous expressions of whole pathways for the production of compounds is possible [6]. Enzyme mechanisms can be addressed through structure-based site-directed mutagenesis, which may also lead to novel products [7]. An alternative approach is offered by computational chemistry, which is ideally performed in combination with experimental verification of the computational results, e.g., through the enzymatic conversion of isotopically labelled compounds [8].

This thematic issue will cover all different aspects of studying the roles of enzymes in the biosynthesis of natural products. Also contributions showing the application of enzymes in synthetic organic chemistry will be welcome. I am grateful to all colleagues who have contributed to this issue and to the Editorial Team of the Beilstein-Institut for their professional support. I wish the readers of this issue some stimulating new insights into enzyme research in natural product biosynthesis.

Jeroen S. Dickschat

Bonn, August 2022
